# A Case Report of a Patient with Soaring Clozapine Levels after Developing a Urinary Tract Infection

**DOI:** 10.1155/2024/9147674

**Published:** 2024-02-20

**Authors:** Adesh Kumar Agrawal, Soumitra Das, Lorenzo Abednego B. Adre, Nakka Raghuma, Sharanya Kaushik, Adarsha Adhikari

**Affiliations:** ^1^All India Institute of Medical Sciences, Deoghar, India; ^2^Department of Psychiatry, Western Health, Victoria, Australia; ^3^Manila Doctors Hospital, Manila, Philippines; ^4^GSL Medical College and General Hospital, Rajahmundry, India; ^5^Bangalore Medical College and Research Institute, Bengaluru, India; ^6^Gandaki Medical College, Pokhara, Nepal

## Abstract

Clozapine is an antipsychotic medicine used to treat mental illnesses that is resistant to therapy. It can induce dose-dependent adverse effects such as increased susceptibility to infections and hematological irregularities. In this case report, we present a 37-year-old woman with schizoaffective disorder who experienced clozapine side effects following a moderate urinary tract infection (UTI). Her serum clozapine levels and side effects were increased throughout her UTI but resolved once the UTI was managed conservatively. We reviewed clozapine's pharmacokinetic properties to understand why serum levels rose during infection. While we could not definitely explain the mechanism of elevation, we emphasize the importance of monitoring serum clozapine levels and keeping watchful for adverse effects, as well as heightened scrutiny, evaluation for recent infections, and regular monitoring of patients.

## 1. Introduction

Clozapine is commonly prescribed for the treatment of severe mental illnesses that are resistant to other therapies [1, 2]. The desired therapeutic range for clozapine plasma levels typically falls between 350 and 420 *μ*g/L, although it can vary among individual patients. In order to ensure an adequate response and minimize side effects, regular monitoring of serum clozapine levels is crucial. Some side effects, such as seizures, sedation, constipation, dysautonomia, and hyperinsulinemia, are known to be dose-dependent.

Clozapine can induce various hematological changes, including neutropenia, eosinophilia, and thrombocytopenia, which may make the patient more susceptible to both local and systemic infections [2, 3]. Additionally, it is important to note that infections can affect serum clozapine levels by influencing its metabolism [4].

In this context, we present a patient with a mild urinary tract infection (UTI) who concurrently experienced worsening side effects of clozapine. The side effects subsequently improved upon resolution of the UTI along with a subsequent decrease in the serum clozapine levels.

## 2. Case Presentation

### 2.1. Patient Information

A 37-year-old married nonsmoking Indian woman, the mother of a 5-year-old daughter, had been diagnosed with schizoaffective disorder for the last 15 years. The patient had a history of two previous psychiatric hospitalizations because of medication nonadherence but reported to be compliant to medication at the time presented in this report. She had been on a steady dose of clozapine in monotherapy, 200 mg per day for 1 year and her corresponding serum clozapine level was 475 *μ*g/L (norclozapine 390 *μ*g/L). The patient attended the hospital with her husband due to worsening of psychotic symptoms. Family and medical history was inconsequential.

### 2.2. Treatment Modifications

The clozapine dose was optimized to 350 mg/day after her current visit due to worsening of psychotic symptoms. Following the dose adjustment, her clozapine level increased to 666 *μ*g/L (norclozapine 384 *μ*g/L). Subsequent follow-ups revealed the following levels: after 4 weeks of stable dose—726 *μ*g/L (norclozapine 391 *μ*g/L), post 7 weeks—661 *μ*g/L (norclozapine 437 *μ*g/L), and post 11 weeks—1,409 *μ*g/L (742 *μ*g/L).

### 2.3. Clinical Laboratory Results

At the 11-week mark, additional laboratory investigations showed elevated C-reactive protein (CRP) levels (17 mg/L), an increased erythrocyte sedimentation rate (ESR) (63 mm/hr), a gamma-glutamyl transferase (GGT) level of 70 U/L, a white cell count (WCC) of 14.7 × 10^9^/L, and a neutrophil count of 11.1 × 10^9^/L. Other investigations yielded normal results. The levels of clozapine and other markers have been depicted in Table 1.

### 2.4. Temporary Discontinuation and Resolving Symptoms

The patient reported mild burning micturition at the week 11 mark. Urine microscopy showed increased leukocytes (70 × 10^6^/*µ*L) and erythrocytes (10 RBC/*µ*L). As a result of the elevated clozapine level and the urinary symptoms, clozapine was temporarily discontinued. Considering the mild severity, conservative management of the UTI was implemented. Within 2 weeks, the burning sensation resolved spontaneously, and blood parameters gradually returned to normal. Clozapine was reinitiated at the dosage of 350 mg/day.

### 2.5. Follow-Up and Resolution

In subsequent follow-ups, the patient's most recent serum clozapine level returned to normal at 578 *μ*g/L (359 *μ*g/L), coinciding with the resolution of sedation. The two subsequent readings of her clozapine levels remained within normal limits during the 4-weekly reviews.

## 3. Discussion

Side effects of clozapine can be dependent on doses of clozapine and the duration for which it has been given [3]. Clozapine is nearly completely absorbed from the gut, highly protein bound and has high first-pass metabolism in the liver [5]. It is primarily metabolized by demethylation and oxidation [5]. We reviewed each aspect of the pharmacokinetics of clozapine to find the cause of raised serum clozapine level during periods of even mild infection. First, the importance of P-glycoprotein in clozapine absorption during inflammation is uncertain. While it usually aids intestinal clozapine uptake, studies conflict on its expression during inflammation. More research is needed to clarify its role when systemic inflammation is present [6].

Second, clozapine is highly bound to the acute-phase protein alpha-1-acid glycoprotein, which increases during inflammatory conditions. According to this, low-serum clozapine level is expected, which does not fit with the raised clozapine levels in our case [7]. During infection, elevated alpha-1-acid glycoprotein may increase total clozapine levels while mitigating side effects by binding more drug. If only free clozapine elicits effects, total levels alone may not correlate with adverse events experienced. This pharmacokinetic relationship merits consideration when managing clozapine during infection, as bound drug does not indicate active concentrations. More research is needed to clarify protein binding's clinical implications for dosing and tolerability.

However, a systematic review proposed that the potential inhibition of cytochrome P450 enzymes CYP1A2, CYP2C19, and CYP3A4, by inflammatory mediators like interleukin-6 (IL-6), tumor necrosis factor-alpha (TNF-*α*), interferon, and CRP results in decreased clozapine metabolism and an increased clozapine : norclozapine ratio [4].

Finally, with regard to the metabolism of clozapine, a surge in various inflammatory markers, including TNF-*α*, IL-6, and CRP is seen during inflammation and these markers inhibit the expression of CYP1A2, i.e., increase the serum clozapine level [8, 9]. No additional drugs were prescribed to treat UTI in our patient; hence drug–drug interactions were unlikely.

Our patient had a mild infection, showed a significant increase in clozapine levels, and developed clozapine side effects (sedation). It is imperative that during the COVID-19 pandemic, cases of clozapine toxicity were found [10]. A systematic review also showed the rise in serum clozapine levels in various infectious and inflammatory conditions [4]. Hence, it is essential to measure serum clozapine levels and monitor the side-effects of clozapine more frequently when the patient has infectious and inflammatory conditions. It is essential to understand the complexity of prescribing clozapine to patients with schizophrenia because (1) there is evidence of the increased acute phase reactant in schizophrenia, and (2) schizophrenic patients are more prone to develop various infections. Therefore, from the clinical point of view, it is pivotal to be alerted to do regular serum clozapine monitoring and ask for recent infection history if the patient shows clozapine side effects on a stable dose.

## 4. Conclusion

The changes in clozapine levels seen in this case suggest that even mild infections can alter clozapine metabolism and clearance, resulting in higher medication levels and related side effects. These findings highlight the significance of regular monitoring of serum clozapine levels and enhanced awareness for infections in clozapine therapy patients, particularly those with schizophrenia who may be more susceptible to infections. Clinicians should evaluate clozapine toxicity in patients who report unexplained worsening of symptoms when on a steady dose of the medicine, particularly if the patient has infectious or inflammatory diseases. More research is needed to determine the precise mechanisms underlying the combination of clozapine, infections, and alterations in medication metabolism. Meanwhile, vigilant monitoring and timely management of infections in clozapine patients are critical for optimizing treatment outcomes and preserving patient safety.

## Figures and Tables

**Table 1:** The patient's levels of clozapine and other markers were monitored over the course of several weeks.

Date	Reference interval	Baseline	Week of Presentation	Week 4	Week 7	Week 11	Week 12	Week 14	Week 18
Dose of clozapine	200–450 mg/day	200	350	350	350	350	0	350	350
Clozapine concentrations	350–420 *μ*g/L	475	666	726	661	1409	—	578	598
Norclozapine concentrations	—	390	384	391	437	742	—	359	373
CRP	<10 mg/L	—	—	—	—	17	—	9	—
ESR	<30 mm/hr	—	—	—	—	63	—	23	—
WBC	4.5–11.0 × 10^9^/L	—	—	—	—	14.7 × 10^9^/L	—	4.7 × 10^9^/L	—
Neutrophils	2–7.5 10×^9^/L	—	—	—	—	11.1 × 10^9^/L	—	2.1 × 10^9^/L	—
GGT	<30 IU/L	—	—	—	—	70 IU/L	—	26 IU/L	—
Urine leukocytes	0–5 × 10^6^/*µ*L	—	—	—	—	70 × 10^6^/*µ*L	—	3 x 10^6^/*µ*L	—
Urine erythrocytes	0–5 RBC/*µ*L	—	—	—	—	10 RBC/*µ*L	—	2 RBC/*µ*L	—
Side-effects	—	—	—	—	—	Mild sedation	—	—	—
Somatic symptoms	—	—	—	—	—	Mild burning micturation	—	—	—

*Abbreviations*. CRP, C-reactive protein; ESR, erythrocyte sedimentation rate; WBC, white blood corpuscles; GGT, gamma-glutamyl transferase; IU, international units; RBC, red blood corpuscles.

## Data Availability

Data for this study are not declared.

## Additional Points

*Limitations*. Limitations of this case report include the lack of medication supervision, making it uncertain if the patient consistently took the same dose of clozapine. The possibility of the patient using other medications, including over-the-counter drugs, which could impact clozapine's pharmacokinetics, was not addressed. Changes in caffeine consumption or other dietary factors during the UTI were not documented, potentially confounding the interpretation of clozapine metabolism. These limitations underscore the need for further research and highlight potential confounding factors that may have influenced the observed results.
